# Mapping the distribution of Lyme disease at a mid-Atlantic site in the United States using electronic health data

**DOI:** 10.1371/journal.pone.0301530

**Published:** 2024-05-31

**Authors:** Paul M. Lantos, Mark Janko, Lise E. Nigrovic, Felicia Ruffin, Takaaki Kobayashi, Yvonne Higgins, Paul G. Auwaerter

**Affiliations:** 1 Duke University School of Medicine, Durham, NC, United States of America; 2 Duke Global Health Institute, Durham, NC, United States of America; 3 Boston Children’s Hospital, Boston, MA, United States of America; 4 University of Iowa Hospital and Clinics, Iowa City, IA, United States of America; 5 Sherrilyn and Ken Fisher Center for Environmental Infectious Diseases, Johns Hopkins University School of Medicine, Baltimore, MD, United States of America; Pacific Northwest National Laboratory, UNITED STATES

## Abstract

Lyme disease is a spatially heterogeneous tick-borne infection, with approximately 85% of US cases concentrated in the mid-Atlantic and northeastern states. Surveillance for Lyme disease and its causative agent, including public health case reporting and entomologic surveillance, is necessary to understand its endemic range, but currently used case detection methods have limitations. To evaluate an alternative approach to Lyme disease surveillance, we have performed a geospatial analysis of Lyme disease cases from the Johns Hopkins Health System in Maryland. We used two sources of cases: a) individuals with both a positive test for Lyme disease and a contemporaneous diagnostic code consistent with a Lyme disease-related syndrome; and b) individuals referred for a Lyme disease evaluation who were adjudicated to have Lyme disease. Controls were individuals from the referral cohort judged not to have Lyme disease. Residential address data were available for all cases and controls. We used a hierarchical Bayesian model with a smoothing function for a coordinate location to evaluate the probability of Lyme disease within 100 km of Johns Hopkins Hospital. We found that the probability of Lyme disease was greatest in the north and west of Baltimore, and the local probability that a subject would have Lyme disease varied by as much as 30-fold. Adjustment for demographic and ecological variables partially attenuated the spatial gradient. Our study supports the suitability of electronic medical record data for the retrospective surveillance of Lyme disease.

## Introduction

Lyme disease is a spatially heterogeneous tick-borne infection caused by the spirochete *Borrelia burgdorferi* and transmitted by *Ixodes* spp. ticks. Approximately 85% of all cases in the United States occur in the mid-Atlantic and northeastern states [1] particularly from northern Virginia through northern New England. The risk of human Lyme disease appears to decline along a north-south gradient within Virginia, and this is corroborated by a decreased abundance *B*. *burgdorferi*-infected *Ixodes scapularis* nymphs in this region [2, 3]. In a large tick survey of Lyme disease risk in the eastern United States, the abundance of *B*. *burgdorferi*-infected *I*. *scapularis* nymphs appeared to drop south of the 39^th^ latitudinal parallel, which passes through Maryland between Baltimore and Washington, DC [2].

The geographic range of Lyme disease cases appears to be expanding, however, including southwards into southern Virginia and North Carolina [4–7]. Epidemiologic studies of Lyme disease are necessary to understand its potential geographic range. However, these studies rely upon passive public health case reporting and entomologic surveillance for the causative agent and its tick vector, both of which have limitations.

Case reporting of Lyme disease is limited by surveillance definitions that do not entirely correspond to clinical diagnoses made in practice, inconsistent ability to exclude cases imported from other regions, and underreporting, which is a well-documented problem [8, 9]. In Maryland, various studies using different data sources has produced a somewhat inconsistent picture of where Lyme disease incidence is highest, with some publications differing from contemporaneous public health data [10–14]. Studies of *B*. *burgdorferi-*infected *Ixodes* spp. nymphs are our best ecologic correlate of human risk [2, 3]. Several factors, however, limit their utility in Lyme disease epidemiology, including the efforts and expense needed to collect and test ticks, limiting the scale on which such studies can be done. Moreover, tick collection studies are primarily performed on public lands, which may not correspond well to where most people are exposed to infection, as well as on types of terrain that most people at risk do not frequent.

An alternative method that may have utility for disease surveillance is the geospatial analysis of medical record data. Patient care generates extensive clinical data that is now accessible through electronic medical record (EMR) queries. It is possible to use medical record data in spatial epidemiology research when combined with geographic identifiers and spatial analytical methods. In this study, we report a geospatial analysis of electronic medical record data from the Johns Hopkins Health System, which serves the city and environs of Baltimore, Maryland, USA, within the mid-Atlantic region. Our statistical hypothesis was that the probability of Lyme disease is geographically heterogeneous within the Baltimore metropolitan area. Our overall intent was to assess the suitability of EMR-based spatial analysis to study the epidemiology of Lyme disease at this geographic scale.

## Materials and methods

This study was approved by the Institutional Review Boards of Johns Hopkins University School of Medicine (Baltimore, Maryland, USA) and the Duke University Health System (Durham, North Carolina, USA). Duke served as the analytical and statistical center for this study. Informed consent and HIPAA authorization were waived for this retrospective study.

Our approach was to perform a case-control spatial statistical analysis of Lyme disease risk as a function of geography. The data sources were individual electronic medical records, from which we obtained the geographic coordinates of their home address. Spatial regression models, described below, were populated using records from the Johns Hopkins Health System.

The Johns Hopkins Health System includes five hospitals and a primary care and specialty clinic network in Maryland and Washington, DC. We used two patient data sources from the Johns Hopkins Health System for this study: subjects tested for Lyme disease who were identified through a medical records query and confirmatory chart review (hereafter referred to as the “retrospective cohort”); and a previously published cohort of subjects who had been referred for a Lyme disease evaluation (hereafter referred to as the “referral cohort”) [15]. To identify the retrospective cohort, medical records were queried for patients with a positive two-tiered serologic test for Lyme disease who had been tested between January 1, 2005 and December 31, 2014. Data were accessed on July 19, 2016. The medical records of these individuals were reviewed for evidence of a Lyme disease-associated syndrome within 30 days of their diagnostic test based on a finding or diagnosis of meningitis, 7th cranial nerve palsy, neuroborreliosis, AV nodal block, carditis, or arthritis. Searching using diagnostic codes did not yield additional records. The goal, as such, was not necessarily to identify every person with Lyme disease but rather to have a cohort in which all subjects had a high likelihood of Lyme disease because of well-documented objective findings. Subjects with a syndrome compatible with Lyme disease at the time when their test was performed were retained as our retrospective cohort for this study.

The referral cohort was comprised of subjects who had been referred to Johns Hopkins for a Lyme disease evaluation between January 1, 2000 and December 31, 2013. After a record review by study personnel, these subjects were categorized as having active Lyme disease, past (resolved) Lyme disease (which had to include at least two years of interval wellness after Lyme disease diagnosis), possible Lyme disease, or not Lyme disease for those who lacked clinical or serologic evidence of Lyme disease. Categories and methods were based on well-established clinical and surveillance descriptions [16, 17]. The referral dataset is described in greater detail in previously published studies [15, 18]. Individuals were excluded from the retrospective cohort if they already had been identified in the referral cohort.

For a control population, we used referral subjects who had been determined not to have Lyme. Thus, the groups we used for our analyses were (1) the retrospective cohort, (2) referral subjects with active Lyme disease, (3) referral subjects with past Lyme disease who had been adjudicated not to have active Lyme disease, and (4) referral subjects without Lyme disease who were analyzed as negative controls. Our subject database included sex and age at the time of their Lyme disease referral (for referral subjects) or test (for retrospective subjects).

We did not create a separate control cohort based solely on subjects whose EMR had a negative Lyme disease test. This is because high volume, low probability Lyme disease testing is commonly performed, particularly in some specialty scenarios (e.g. neurology and rheumatology) and for patients with undifferentiated somatic syndromes. Thus the clinical, demographic, and geographic distribution of negative Lyme disease tests may not adequately correspond to the general underlying population nor to the overall risk of Lyme disease exposure. Creating a suitable negative control dataset from EMR data would have required a more intensive chart-review based study to identify subjects tested for a common set of indications, and this set of subjects could then be dichotomized based on test results. This type of chart review was not possible at the time this study was performed.

All subjects whose residential address was within 200 km of Johns Hopkins Hospital were retained for spatial analysis. Our goal was to predict our model to a 100 km radius around Johns Hopkins Hospital; thus we populated our model with subjects up to 100 km more distant so that the boundary of our prediction area would fall in the middle of our data and not at the very edge. To incorporate key ecological variables in our models, we obtained land cover and hydrogeography data, which have been used in ecoepidemiology studies of Lyme disease [7, 19–22]. We obtained the publicly available 2011 National Land Cover Database, which is a continuous geographic data set that contains 30x30 meter pixels with classified land cover. To simplify our analysis we consolidated forest classifications (deciduous, coniferous, and mixed) into a single forest category, and consolidated woody wetlands and emerging herbaceous wetlands into a single wetlands category. Using zonal statistics in ArcGIS Pro 3.0 (ESRI, Redlands, CA, USA) we calculated the total forest and wetland area within a 1 km radius of each subject’s home. Finally, we computed the distance from each subject’s address to a river or stream in the United States Natural Atlas Water Features dataset (accessed from ESRI within ArcGIS).

For our statistical analyses we fit hierarchical Bayesian spatial models using the brms package [23] in the statistical programming language R (www.r-project.org). The brms package facilitates the construction of Bayesian regression models, which are transferred to the sampling program Stan [24] for sampling the posterior probability distribution. Continuous variables were centered on 0 by subtracting the mean, then standardized by dividing by one standard deviation. Our models used binomial probability functions because of the binary nature of our outcome variable (e.g. Lyme disease vs not Lyme disease). To study the impact of geography we included the longitude and latitude coordinates for each subject’s home address in our models. A 2-dimensional smoothing spline was used, which allowed us to evaluate our models’ outcome variables as a function of continually varying 2-dimensional space. Treating coordinate space as a smoothed rather than a linear variable allowed us to identify clusters or foci where the probability of Lyme disease departed from the average. Smoothing splines were fit by accessing the mgcv package in R [25] from within brms.

We ran the following models: active Lyme disease patients versus non-Lyme disease controls, both from the referral dataset; Lyme disease patients from the diagnostic testing query versus controls from the referral dataset; and individuals with past Lyme disease versus non-Lyme disease controls, both from the referral dataset. We also constructed an unadjusted model of active versus past Lyme disease patients to evaluate whether these two groups had different geographic distributions than one another. The units of analysis in our models were individual patients, dichotomized by whether they had Lyme disease or not. Our primary predictor variable was a 2-dimensional spline smooth of each subject’s home address, which was encoded as longitude-latitude coordinate pairs. Smoothing functions allow us to evaluate the local variability of the outcome variable within the range of the smoothed variable, in this case, the 2-dimensional coordinate pairs. We modeled the odds of a positive Lyme disease diagnosis over a 2-dimensional coordinate space. For comprehensibility, we converted odds to probability.

The aforementioned models were unadjusted, evaluating only Lyme disease as a function of geography. For the two models comparing active Lyme disease cases to controls (the active referral subjects and the testing query subjects), we also ran adjusted models incorporating age, sex, land cover, and proximity to water as linear predictors. We used normal distributions with mean 0 and standard deviation 1 as regularizing priors for all linear predictors as well as the model intercept. The output of our adjusted models was expressed as odds ratios (OR). For numerical variables (e.g. age) this can be interpreted as the OR of Lyme disease for each 1 standard deviation increase. For sex, which was our one categorical variable, this should be interpreted as the OR of Lyme disease for males compared with the reference group females.

**Fig 1:**
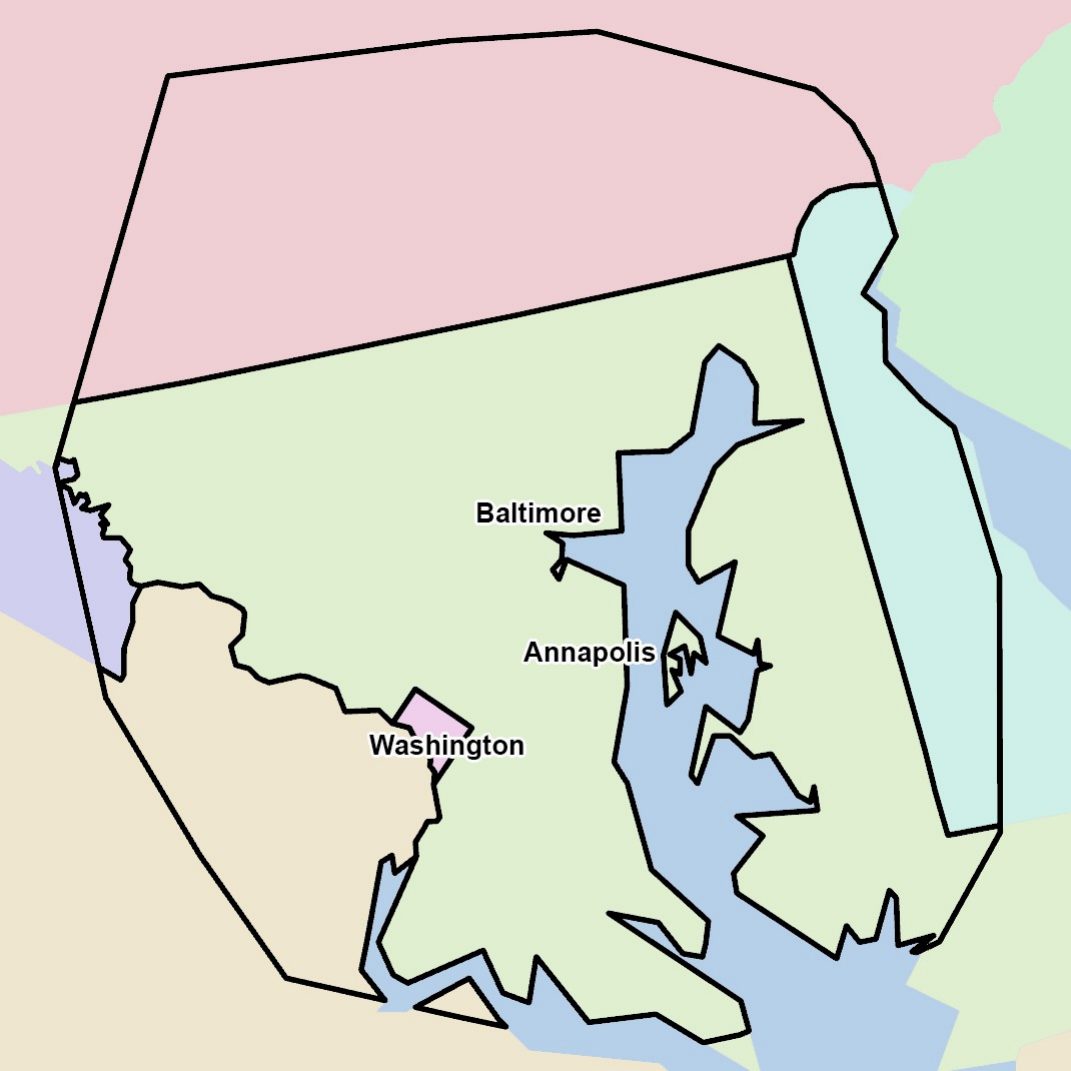
Geographic study range. This irregular polygon defines a roughly 100 km zone around Baltimore, Maryland, USA. Models were constructed using subject data from a larger, 200 km radius and then predicted into this 100 km zone.

After fitting each model, we predicted the probability of Lyme disease onto a dense longitude-latitude grid covering an area approximately 100 km from Johns Hopkins Hospital (**Fig 1**). This allowed us to create a Lyme disease risk map, in which the probability of Lyme disease was displayed geographically. Additionally, this allowed us to adjust for the influence of predictors, such as land cover. We predicted our models to a smaller geographic area (100 km) than the area from which we sampled our subjects (200 km) for two reasons: first, subject data would inform the boundaries of our prediction grid from well beyond the edge of the grid; and second, because with increasing distance the density of our subjects rapidly decreased, which in turn reduced confidence in any prediction. We used contours to circumscribe geographic regions with a 90, 95, and 99.5% likelihood that the local probability differed from the global probability. The background color of the resulting maps signifies the local probability of Lyme disease, and the contours represent the probability that the area circumscribed differs from the average–this can be interpreted as an expression of confidence or significance. For the non-spatial individual covariates we report their effect size as odds ratios (OR), and uncertainty is reported as 95% credible intervals. In this Bayesian context the 95% credible interval represents the upper and lower values between there is a 95% probability of finding a parameter’s true estimate. Thus it can be interpreted as the estimate’s precision.

To compare our models against publicly available surveillance data from the same region and time period we obtained annual confirmed and probable case counts of Lyme disease for each Maryland county beginning in 2008 and ending in 2019 [11]. Probable and confirmed cases were as determined by CDC surveillance criteria. To assess if there was any significant spatial change in case distribution over time, we computed each county’s case incidence per 100,000 population for the three year intervals of 2008–2010, 2011–2013, and 2014–2016. We used United States Census annual population estimates for the final year of each time interval (2010, 2013, and 2016) as a population reference for each county [26].

## Results

Our dataset included 148 subjects with Lyme disease from the retrospective cohort. The referral cohort included 177 subjects with active Lyme disease and 838 subjects without Lyme disease who were included as controls. Additionally, there were 146 subjects with prior Lyme disease in the referral cohort. The age and sex distribution of our cohorts are presented in **Table 1**.

**Table 1:** Demographics of the study groups. The “retrospective” cohort was those subjects who had both positive Lyme disease tests and contemporaneous clinical documentation supporting that diagnosis. The “referral” cohort included individuals who had been referred for a Lyme disease evaluation, and were further categorized into Active, Past, or Negative (Non-Lyme disease) disease groups based on records review. The referral subjects who were negative were used as control subjects for our analyses.

	Retrospective		Referral	
		Active Lyme Disease	Past Lyme Disease	Negative (Control group)
Number	148	177	146	838
% Women	38.5	48.6	50.1	66.8
Mean Age	18.1	44.6	48.1	44.2

**Fig 2:**
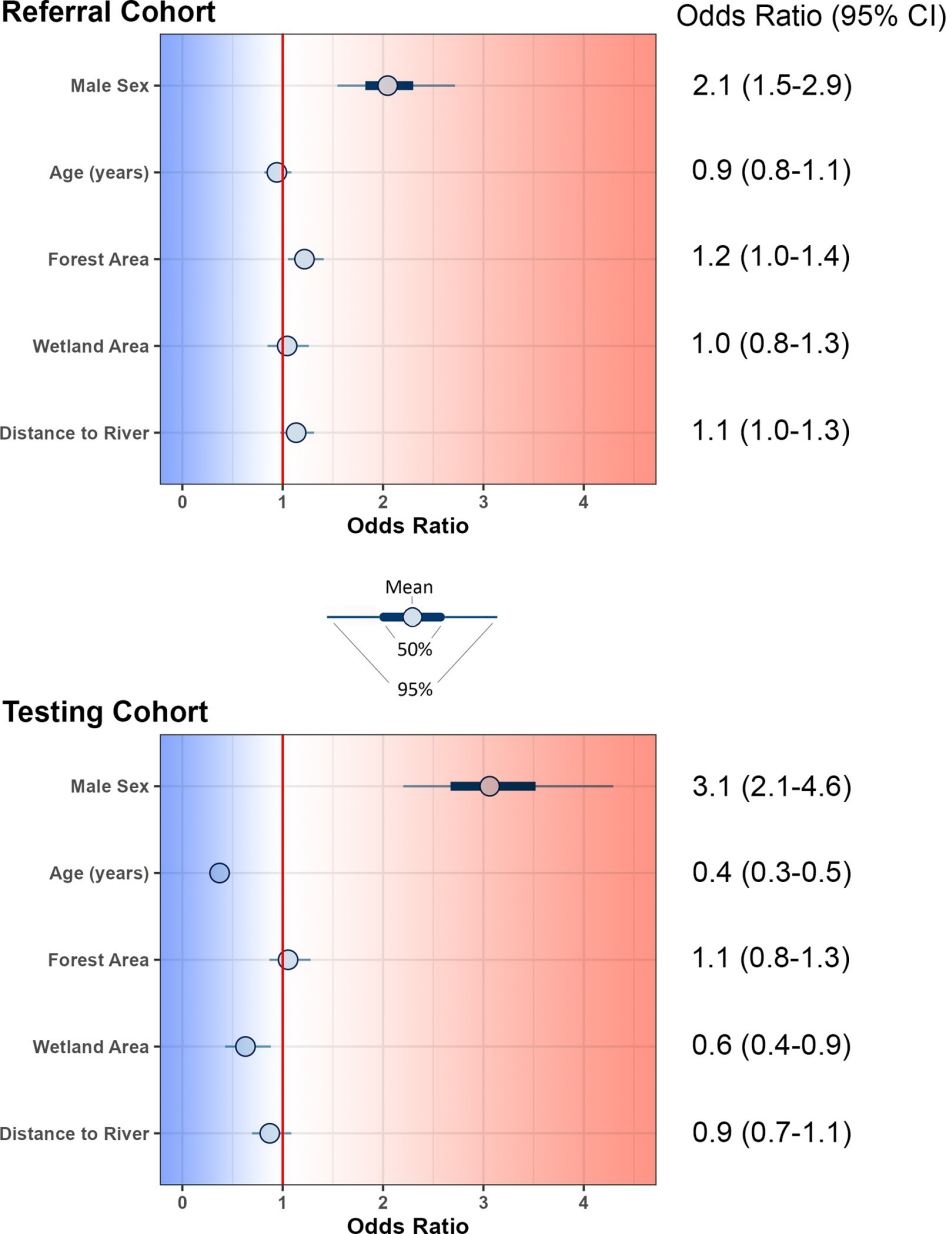
Covariates associated with Lyme disease in referral subjects with active Lyme disease (top) and retrospective subjects (bottom). The x-axis represents the odds ratio (OR) of Lyme disease for each 1 standard deviation increase in the continuous variables labeled on the y axis. In the case of sex the OR represents the OR of Lyme disease among male vs female subjects. In the case of age in the retrospective cohort, a 1 standard deviation increase in age is associated with an OR of 0.4 –in other words, higher age is negatively associated with Lyme diseaseBars represent the 50% (thick) and 95% (thin) credible intervals, and circles represent the mean. Any parameter that does not overlap 0 has at least a 95% probability of being associated with Lyme disease.

Compared with our control subjects, subjects with Lyme disease were much more likely to be male (**Fig 2**, referral cohort OR 2.1, 95% CI 1.5–2.9, retrospective cohort OR 3.1, 95% CI 2.1–4.6). Younger age was associated with Lyme disease in our retrospective cohort (OR 0.4, 95% 0.3–0.5) but not among referral subjects (OR 0,9, 95% CI 0.8–1.1). Environmental variables were inconsistently associated with Lyme disease cases, and the effect size was generally low. For the referral cohort, greater forest area (OR 1.2, 95% CI 1.0–1.4) and greater distance from a river (OR 1.1, 95% CI 1.0–1.3) were associated with a marginally higher odds of Lyme disease. For the retrospective cohort, wetland area was inversely associated with Lyme disease risk (OR 0.6, 95% CI 0.4–0.9), but other environmental variables were not (**Fig 2**).

**Fig 3:**
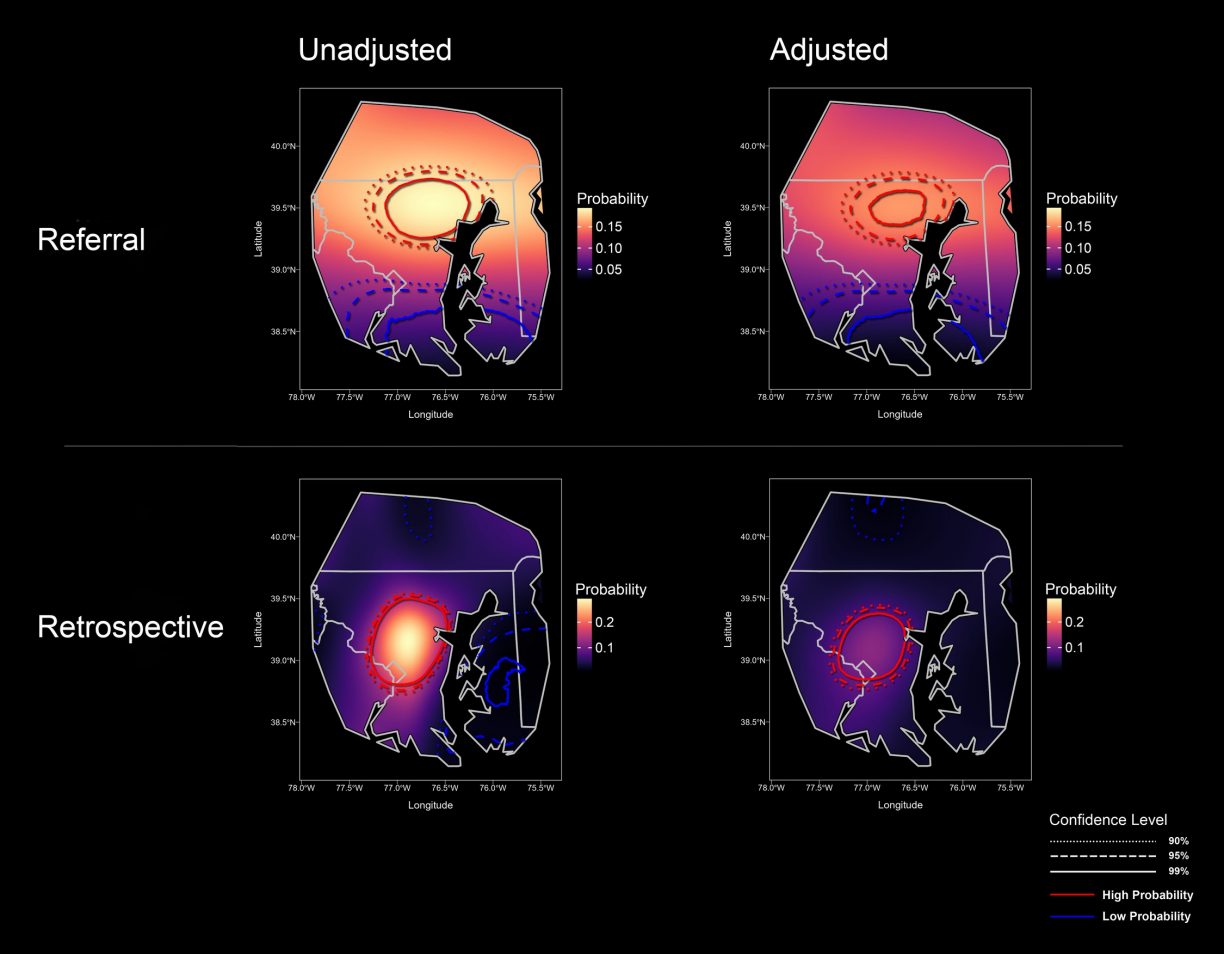
Unadjusted and adjusted maps of the predicted probability of Lyme disease. The color gradient represents the computed probability of Lyme disease. Red contours represent higher than average probability and blue contours represent lower than average probability, with the 90%, 95%, and 99% confidence levels denoted by dotted, dashed, and solid lines respectively. In this Bayesian analysis confidence contours encircle to “credible” or probability intervals, i.e. areas where there is a 90%, 95%, or 99% probability that the area encircled differs from the overall mean. The probability of Lyme disease in the active Lyme disease referral cohort was greatest in the northern suburbs of Baltimore and northern Maryland / southern Pennsylvania, USA. The probability was lowest in southern Maryland and northeastern Virginia. In the retrospectivecohort, the probability of Lyme disease was greatest to the west of Baltimore. Adjustments for ecological and demographic variables attenuated the probability range.

**Fig 4:**
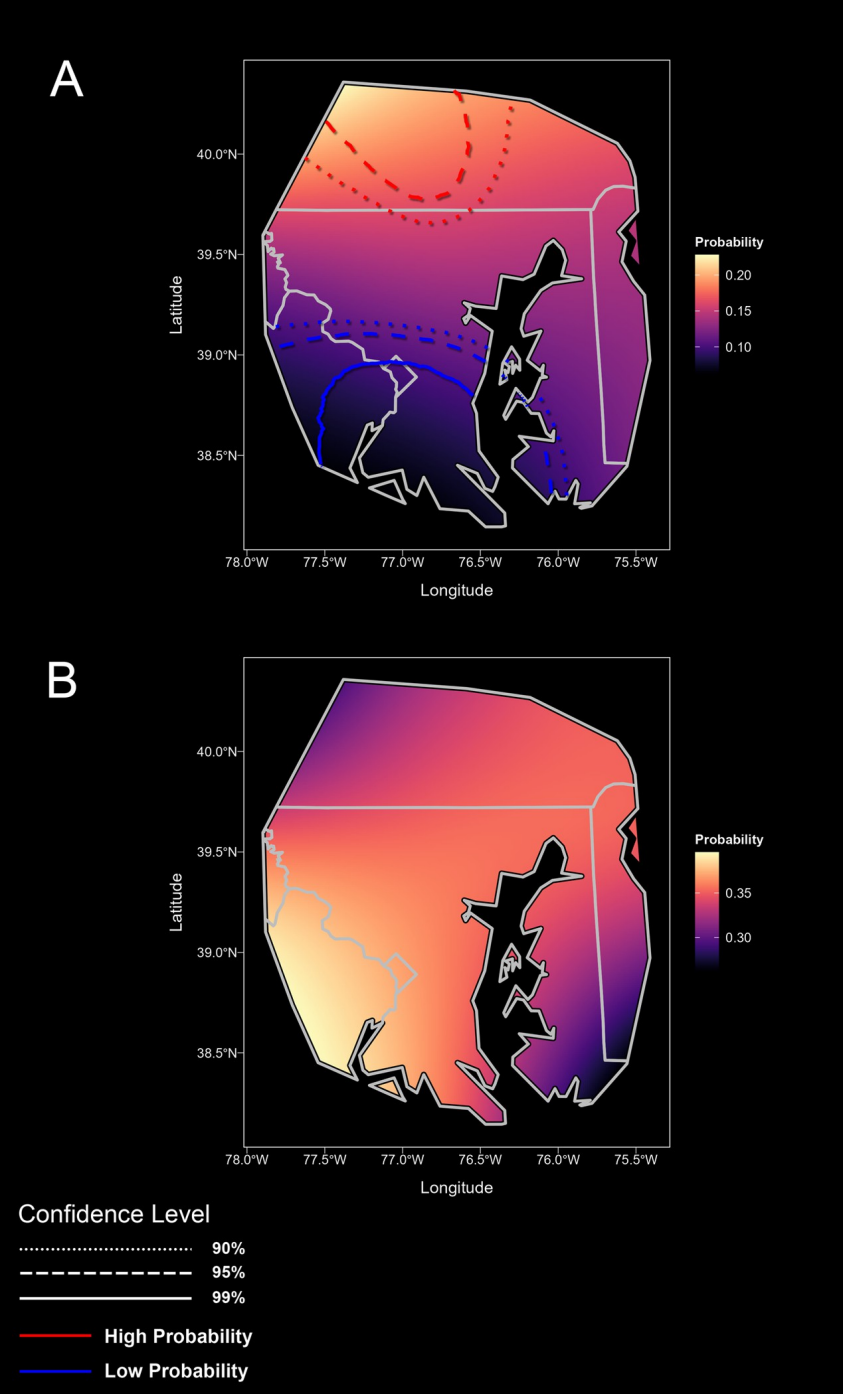
**A**–Distribution of past Lyme disease compared with negative controls. The probability of a history of Lyme disease was highest in the northern part of the study zone in Maryland, USA, and decreased along a north-south gradient. **B**–Comparison of active to past Lyme disease subjects from the referral cohort. There was no statistically apparent difference in the distribution of subjects with active versus past Lyme disease.

The probability of Lyme disease was spatially variable (**Fig 3**). Our unadjusted model of referral subjects with active Lyme disease exhibited a marked north-south gradient across the study area. The probability of Lyme disease decreased from 0.2 in the highest prevalence areas to 0.03 in the lowest. Spatial heterogeneity was also seen for the retrospective cohort, with the probability of Lyme disease ranging from 0.01 to 0.3. Still, the highest risk area was concentrated to the west of Baltimore. In both the referral and the testing models, we identified areas of both significantly higher and lower than average probability of Lyme disease. Adjustment for environmental and demographic partially attenuated the observed spatial heterogeneity, but overall the distribution of high vs low risk areas remained unchanged after adjustment. Referral subjects with past Lyme disease were also distributed along a north-south gradient compared with negative control cases (**Fig 4**). There was no significant spatial difference identified between the active Lyme referral cohort and the past Lyme referral cohort.

## Discussion

In a novel spatial analysis of patient referral data, we have identified substantial geographic heterogeneity in the probability of Lyme disease around Baltimore, Maryland. Within a 100 km radius, we observed as much as a 30-fold range in the probability of Lyme disease, with particularly high-risk areas to the north and west of Baltimore. These suburban and rural areas have a well-established risk of Lyme disease. The probability of past Lyme disease was distributed more diffusely but followed a similar north-south gradient. Insight into a patient’s geographic exposure, even within the catchment of a single medical center, can give inference into the pre-test probability of Lyme disease.

**Fig 5:**
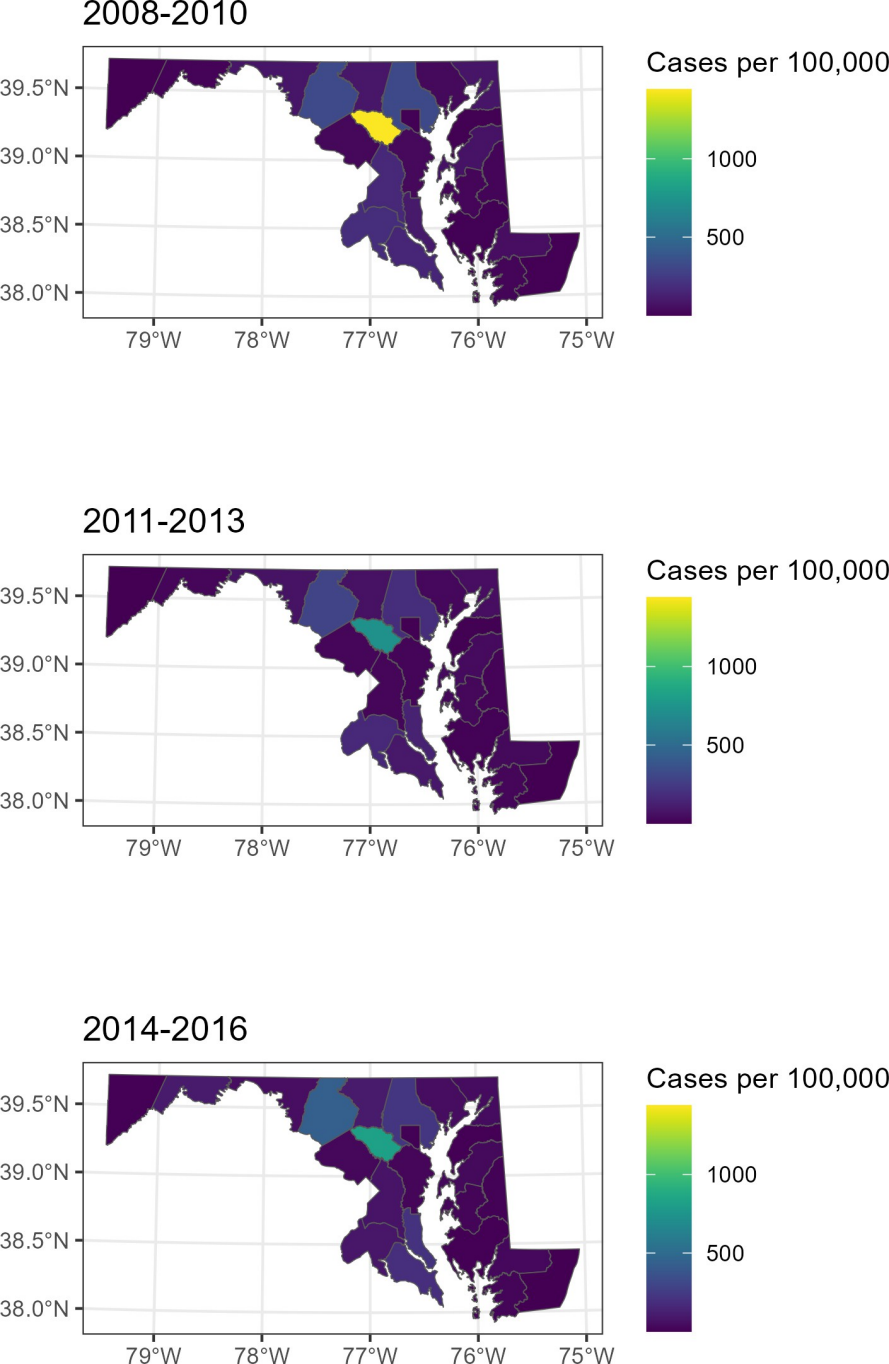
Maryland county-based Lyme disease incidence data from 2008–2016, divided into 3 year intervals. Lyme disease case data comes from public health surveillance data from the Maryland Department of Health, and population data from the United States Census. The geographic distribution of Lyme disease remained relatively constant over the study period.

The distribution of Lyme disease we observed was consistent with public health surveillance data from this area (presented in Fig 5), which shows a proportionally higher incidence of Lyme disease to the west and northwest of Baltimore that has persisted across the duration of this study, particularly in Howard County [11]. In fact, as a result,this particular county has been the subject of dedicated study [27]. On the other hand, several older studies identified eastern Maryland as a particularly high incidence focus [12, 13, 28, 29]. A more recent study using Medicaid data also identified eastern Maryland as the region with highest risk, but in contrast to older studies western counties had become a second focus [14]. One 1995 study that was limited to Baltimore County identified an increased risk of Lyme disease to the north and northwest of Baltimore City, which is consistent with the distribution we report, but this study evaluated a comparatively small geography [10].The discrepancy between these older studies and ours is likely due to changing epidemiology, but may also result from differences in methodology and data sources. For instance, our study relatively undersampled the eastern reaches of Maryland, which tend to fall in the catchment of other health systems. On the other hand Rebman 2018, by using Medicaid data, had a more spatially unbiased data capture, but may have undersampled more affluent suburban areas where Lyme disease transmission often occurs at the suburb-forest interface.

The lower probability of Lyme disease in southern parts of the study area is most likely related to the diminishing abundance of infected, host-seeking tick vectors in the southern reaches of our study area [2, 29]. A large contemporaneous entomologic study reported that questing *B*. *burgdorferi* s.s.-infected *Ixodes scapularis* nymphs were rarely found south of the 39^th^ parallel, which runs between Baltimore and Washington, DC [3]. The risk of human Lyme disease is correspondingly lower; for instance, according to 2016 estimates from the Centers for Disease Control and Prevention, the incidence of Lyme disease in Maryland was nearly twice that of Virginia (21.2 vs. 11.6 cases per 100,000) [30]. At the same time, there is heterogeneity of both tick abundance and human cases at the sub-state level, and the distribution of human cases has expanded over time [4, 6].

Our adjusted models incorporated both individual and environmental covariates. In both the referral and retrospective cohorts, Lyme disease was associated with male sex. Age was not associated with Lyme disease in the referral cohort. It was associated with Lyme disease in the retrospective cohort, but this is certainly because the referral cohort (from which the controls were derived) did not contain any pediatric subjects. In our study we did not find consistent associations between environmental variables and the risk of Lyme disease. In previous studies proximity to forest, fragmented forest, and forest edge habitat have been consistently identified as risk factors for Lyme disease [10]. These may have been less prominent in our study because a considerable proportion of the population studied resides in urban or peri-urban areas that have comparatively little forest area. Other variables such as climate and soil conditions have been associated with Lyme disease in previous studies; evaluating such variables may be relevant to future directions of our research.

Our study demonstrates the utility of clinical data as a surveillance tool for Lyme disease, which could be a valuable addition to much more labor- and resource-intensive means of surveillance, such as public health case reporting and entomologic studies. Secondary analysis of various data sources has proven to be a valuable addition to understanding Lyme disease epidemiology [31, 32]. Applying geospatial statistical analyses to data from a health system’s electronic medical records may illuminate local disease distribution within that health system’s catchment.

Our study must be interpreted in the context of its limitations. First, we were unable to include subjects with the earliest presentations of Lyme disease (i.e. an erythema migrans skin lesion), because patients with cutaneous Lyme disease can be diagnosed without serologic testing, are usually seen in primary care doctor’s offices rather than referral centers, and do not require specialist care. Second, the different sampling methodologies and source populations for our study cohorts led to demographic differences between them (for instance, age was lower among the retrospective subjects than the referral subjects). Third, the time periods differed slightly between referral and retrospective subjects, though the impact of this is likely minimal given their substantial temporal overlap and the stable geotemporal distribution of Lyme disease during this period (Fig 5). Fourth, the threshold to refer to a specialist academic center may differ with distance the patient must travel, introducing a potential spectrum bias by site of care. Fifth, the referral cases had a less strict definition of Lyme disease than the retrospective cases, which required *a priori* positive serologic testing and a well-documented syndrome. Sixth, we were limited to available medical documentation, which was not collected uniformly for all subjects. Seventh, the residential address may not represent the site of tick exposure, although the majority of Lyme disease cases are known to be acquired near home.

Our spatial inferences are limited by the fact that our retrospective cases and our negative control cases came from different spatial sampling methodologies. The retrospective cohort was collected from a medical records query and review. In contrast, the controls came from a clinic referral cohort, some of whom may have had laboratory results and medical records from unaffiliated sites that would not have been visible via the retrospective query.. Some subjects lived within the catchments of other tertiary referral centers, and individuals who were referred to JHHS from those areas may be biased by unmeasured clinical or insurance factors. This bias is certainly not spatially uniform either. Overall this may produce a spatially heterogeneous spectrum bias.–for example, in the direction of Washington, DC there are other tertiary care health systems quite close to Baltimore, whereas this is not true to the northeast of Baltimore.

The epidemiology of Lyme disease is changing over time, and it is uncertain how generalizable our study is outside the dates within which the primary data was collected. However, our study demonstrates the viability of electronic medical record data as a surveillance tool to contribute to our understanding. Entomologic data on the distribution of infected ticks is critical to understanding Lyme disease epidemiology. Ideally, future studies will join entomologic findings with patient-based data for a richer analysis of geographic risk.

## Conclusions

Our study contributes to understanding the epidemiology of Lyme disease in the Baltimore, Maryland area, a geographic region where Lyme disease transmission appears to be growing. We have shown a strategy for future Lyme disease epidemiology studies using electronic medical record data.

## Supporting information

S1 File (PDF)
