# Supplementary material for: Mapping the distribution of Lyme disease at a mid-Atlantic site in the United States using electronic health data
Source: PLoS One. 2024 May 31;19(5):e0301530. doi: 10.1371/journal.pone.0301530 (PMC11142662; doi:10.1371/journal.pone.0301530)
Supplement: S1 File — (PDF) [file pone.0301530.s001.pdf]

Lyme Disease Study Markdown

2022-09-20

```
library(brms)
library(sf)
library(dplyr)
library(ggplot2)
library(viridis)


prior1 <-     c( prior( normal( 0 , 1 ) , class=b , coef = z.Forest ) ,
                 prior( normal( 0 , 1 ) , class=b , coef = z.Wetland ) ,
                 prior( normal( 0 , 1 ) , class=b , coef = z.River ) ,
                 prior( normal( 0 , 1 ) , class=b , coef = z.Age ) ,
                 prior( normal( 0 , 1 ) , class=b , coef = SexM ) ,
                 prior( normal( 0 , 1 ) , class=Intercept ) )

prior0 <-     c( prior( normal( 0 , 1 ) , class=Intercept ) )

### Consults: Active Lyme vs Not Lyme
consults_unadj <- brm( LymeStatus ~ t2(Longitude, Latitude , k=16) ,
                     prior=prior0 ,
                     data=consults,
                     iter = 6000,
                     control = list( adapt_delta = 0.99 , max_treedepth=20 ) ,
                     family = "Bernoulli" ,
                     chains = 4 ,
                     cores=4 )

consults_adj <- brm( LymeStatus ~ t2(Longitude, Latitude , k=16) + Sex + z.Age +
                       z.River + z.Forest + z.Wetland,
                     prior=prior1 ,
                     data=consults,
                     iter = 6000,
                     control = list( adapt_delta = 0.99 , max_treedepth=20 ) ,
                     family = "Bernoulli" ,
                     chains = 4 ,
                     cores=4 )

### Gradient: Cases vs Consult Controls
gradient_unadj <- brm( LymeStatus ~ t2(Longitude, Latitude , k=16) ,
                prior=prior0 ,
                data=combined,
                iter = 6000,
                control = list( adapt_delta = 0.99 , max_treedepth=20 ) ,
                family = "Bernoulli" ,
                chains = 4 ,
                cores=4 )
```

```
gradient_adj <- brm( LymeStatus ~ t2(Longitude, Latitude , k=16) + Sex + z.Age +
                       z.River + z.Forest + z.Wetland,
                       prior=prior1 ,
                       data=combined,
                       iter = 6000,
                       control = list( adapt_delta = 0.99 , max_treedepth=20 ) ,
                       family = "Bernoulli" ,
                       chains = 4 ,
                       cores=4 )

### Past Lyme vs Controls
pastfit <- brm( LymeStatus ~ t2(Longitude, Latitude , k=16) ,
             prior=prior0 ,
             data=consults_past,
             iter = 6000,
             control = list( adapt_delta = 0.99 , max_treedepth=20 ) ,
             family = "Bernoulli" ,
             chains = 4 ,
             cores=4 )

### Active Lyme vs Past Lyme
lymefit <- brm( LymeStatus ~ t2(Longitude, Latitude , k=16) ,
             prior=prior0 ,
             data=consults_lyme,
             iter = 6000,
             control = list( adapt_delta = 0.99 , max_treedepth=20 ) ,
             family = "Bernoulli" ,
             chains = 4 ,
             cores=4 )



##Consult Maps
pred.u_consults <- as.data.frame( cbind( grid1[,1:2] ,
  fitted(consults_unadj, grid1, allow_new_levels = TRUE)))
pred.a_consults <- as.data.frame( cbind( grid1[,1:2] ,
  fitted(consults_adj, grid1, allow_new_levels = TRUE)))
pred.u_consults$Probability =
  pred.u_consults$Estimate / (1 + pred.u_consults$Estimate)
pred.a_consults$Probability =
  pred.a_consults$Estimate / (1 + pred.a_consults$Estimate)
range(pred.u_consults$Probability)
range(pred.a_consults$Probability)

fv_con.u <- fitted( consults_unadj , newdata=grid1 ,
  summary=FALSE , allow_new_levels=TRUE)
m_con.u <- mean( fv_con.u )
pred.u_consults$p <- colMeans( fv_con.u > m_con.u )

fv_con.a <- fitted( consults_adj , newdata=grid1 ,
  summary=FALSE , allow_new_levels=TRUE)
m_con.a <- mean( fv_con.a )
pred.a_consults$p <- colMeans( fv_con.a > m_con.a )
```

```
consults_u<-
  ggplot(pred.u_consults) +
  geom_raster(aes(Longitude, Latitude, fill=Probability)) +
  scale_fill_viridis(option="A" , limits=c(0.0217233 , 0.19385007)) +
  geom_sf(data=hull, fill=NA, cex=2, color="black") +
  geom_sf(data=states , fill=NA, cex=1 , color="gray") +
  theme_black() +
  theme(panel.grid.minor = element_blank() ) +
  theme(panel.grid.major = element_blank() ) +
  geom_contour( aes( x=Longitude , y=Latitude , z=p) , breaks = c( 0.05 ),
                color="Blue" , cex=1.2, lty=3) +
  geom_contour( aes( x=Longitude , y=Latitude , z=p) , breaks = c( 0.025 ),
                color="Blue" , cex=1.2, lty=2) +
  geom_contour( aes( x=Longitude , y=Latitude , z=p) , breaks = c( 0.005 ),
                color="Blue" , cex=1.2, lty=1) +
  geom_contour( aes( x=Longitude , y=Latitude , z=p) , breaks = c( 0.995 ),
                color="Red" , cex=1.2, lty=1) +
  geom_contour( aes( x=Longitude , y=Latitude , z=p) , breaks = c( 0.95 ),
                color="Red" , cex=1.2, lty=3) +
  geom_contour( aes( x=Longitude, y=Latitude , z=p) , breaks = c( 0.975 ),
                color="Red" , cex=1.2, lty=2)

consults_a<-
  ggplot(pred.a_consults) +
  geom_raster(aes(Longitude, Latitude, fill=Probability)) +
  scale_fill_viridis(option="A" , limits=c(0.0217233 , 0.19385007)) +
  geom_sf(data=hull, fill=NA, cex=2, color="black") +
  geom_sf(data=states , fill=NA, cex=1 , color="gray") +
  theme_black() +
  theme(panel.grid.minor = element_blank() ) +
  theme(panel.grid.major = element_blank() ) +
  geom_contour( aes( x=Longitude , y=Latitude , z=p) , breaks = c( 0.05 ),
                color="Blue" , cex=1.2, lty=3) +
  geom_contour( aes( x=Longitude , y=Latitude , z=p) , breaks = c( 0.025 ),
                color="Blue" , cex=1.2, lty=2) +
  geom_contour( aes( x=Longitude , y=Latitude , z=p) , breaks = c( 0.005 ),
                color="Blue" , cex=1.2, lty=1) +
  geom_contour( aes( x=Longitude , y=Latitude , z=p) , breaks = c( 0.995 ),
                color="Red" , cex=1.2, lty=1) +
  geom_contour( aes( x=Longitude , y=Latitude , z=p) , breaks = c( 0.95 ),
                color="Red" , cex=1.2, lty=3) +
  geom_contour( aes( x=Longitude, y=Latitude , z=p) , breaks = c( 0.975 ),
                color="Red" , cex=1.2, lty=2)


##Gradient Maps
pred.u_gradient <- as.data.frame( cbind( grid1[,1:2] ,
  fitted(gradient_unadj, grid1, allow_new_levels = TRUE)))
pred.a_gradient <- as.data.frame( cbind( grid1[,1:2] ,
  fitted(gradient_adj, grid1, allow_new_levels = TRUE)))
pred.u_gradient$Probability =
```

```
  pred.u_gradient$Estimate / (1 + pred.u_gradient$Estimate)
pred.a_gradient$Probability =
  pred.a_gradient$Estimate / (1 + pred.a_gradient$Estimate)
range(pred.u_gradient$Probability)
range(pred.a_gradient$Probability)

fv_gr.u <- fitted( gradient_unadj , newdata=grid1 ,
  summary=FALSE , allow_new_levels=TRUE)
m_gr.u <- mean( fv_gr.u )
pred.u_gradient$p <- colMeans( fv_gr.u > m_gr.u )

fv_gr.a <- fitted( gradient_adj , newdata=grid1 ,
  summary=FALSE , allow_new_levels=TRUE)
m_gr.a <- mean( fv_gr.a )
pred.a_gradient$p <- colMeans( fv_gr.a > m_gr.a )



gradient_u<-
  ggplot(pred.u_gradient) +
  geom_raster(aes(Longitude, Latitude, fill=Probability)) +
  scale_fill_viridis(option="A" , limits=c(0.005069072 , 0.291391905)) +
  geom_sf(data=hull, fill=NA, cex=2, color="black") +
  geom_sf(data=states , fill=NA, cex=1 , color="gray") +
  theme_black() +
  theme(panel.grid.minor = element_blank() ) +
  theme(panel.grid.major = element_blank() ) +
  geom_contour( aes( x=Longitude , y=Latitude , z=p) , breaks = c( 0.05 ),
              color="Blue" , cex=1.2, lty=3) +
  geom_contour( aes( x=Longitude , y=Latitude , z=p) , breaks = c( 0.025 ),
              color="Blue" , cex=1.2, lty=2) +
  geom_contour( aes( x=Longitude , y=Latitude , z=p) , breaks = c( 0.005 ),
              color="Blue" , cex=1.2, lty=1) +
  geom_contour( aes( x=Longitude , y=Latitude , z=p) , breaks = c( 0.995 ),
              color="Red" , cex=1.2, lty=1) +
  geom_contour( aes( x=Longitude , y=Latitude , z=p) , breaks = c( 0.95 ),
              color="Red" , cex=1.2, lty=3) +
  geom_contour( aes( x=Longitude, y=Latitude , z=p) , breaks = c( 0.975 ),
              color="Red" , cex=1.2, lty=2)

gradient_a<-
  ggplot(pred.a_gradient) +
  geom_raster(aes(Longitude, Latitude, fill=Probability)) +
  scale_fill_viridis(option="A" , limits=c(0.005069072 , 0.291391905)) +
  geom_sf(data=hull, fill=NA, cex=2, color="black") +
  geom_sf(data=states , fill=NA, cex=1 , color="gray") +
  theme_black() +
  theme(panel.grid.minor = element_blank() ) +
  theme(panel.grid.major = element_blank() ) +
  geom_contour( aes( x=Longitude , y=Latitude , z=p) , breaks = c( 0.05 ),
              color="Blue" , cex=1.2, lty=3) +
  geom_contour( aes( x=Longitude , y=Latitude , z=p) , breaks = c( 0.025 ),
              color="Blue" , cex=1.2, lty=2) +
  geom_contour( aes( x=Longitude , y=Latitude , z=p) , breaks = c( 0.005 ),
```

```
                 color="Blue" , cex=1.2, lty=1) +
  geom_contour( aes( x=Longitude , y=Latitude , z=p) , breaks = c( 0.995 ),
                 color="Red" , cex=1.2, lty=1) +
  geom_contour( aes( x=Longitude , y=Latitude , z=p) , breaks = c( 0.95 ),
                 color="Red" , cex=1.2, lty=3) +
  geom_contour( aes( x=Longitude, y=Latitude , z=p) , breaks = c( 0.975 ),
                 color="Red" , cex=1.2, lty=2)



###Past Lyme Map
pred.u_past <- as.data.frame( cbind( grid1[,1:2] ,
  fitted(pastfit, grid1, allow_new_levels = TRUE)))
pred.u_past$Probability = pred.u_past$Estimate / (1 + pred.u_past$Estimate)

fv_past <- fitted( pastfit, newdata=grid1 ,
  summary=FALSE , allow_new_levels=TRUE)
m_past <- mean( fv_past )
pred.u_past$p <- colMeans( fv_past > m_past )

past_map <-
  ggplot(pred.u_past) +
  geom_raster(aes(Longitude, Latitude, fill=Probability)) +
  scale_fill_viridis(option="A" ) +
  geom_sf(data=hull, fill=NA, cex=2, color="black") +
  geom_sf(data=states , fill=NA, cex=1 , color="gray") +
  theme_black() +
  theme(panel.grid.minor = element_blank() ) +
  theme(panel.grid.major = element_blank() ) +
  geom_contour( aes( x=Longitude , y=Latitude , z=p) , breaks = c( 0.05 ),
                 color="Blue" , cex=1.2, lty=3) +
  geom_contour( aes( x=Longitude , y=Latitude , z=p) , breaks = c( 0.025 ),
                 color="Blue" , cex=1.2, lty=2) +
  geom_contour( aes( x=Longitude , y=Latitude , z=p) , breaks = c( 0.005 ),
                 color="Blue" , cex=1.2, lty=1) +
  geom_contour( aes( x=Longitude , y=Latitude , z=p) , breaks = c( 0.995 ),
                 color="Red" , cex=1.2, lty=1) +
  geom_contour( aes( x=Longitude , y=Latitude , z=p) , breaks = c( 0.95 ),
                 color="Red" , cex=1.2, lty=3) +
  geom_contour( aes( x=Longitude, y=Latitude , z=p) , breaks = c( 0.975 ),
                 color="Red" , cex=1.2, lty=2)


###Active vs Past Lyme Map
pred_lyme <- as.data.frame( cbind( grid1[,1:2] ,
  fitted(lymefit, grid1, allow_new_levels = TRUE)))
pred_lyme$Probability = pred_lyme$Estimate / (1 + pred_lyme$Estimate)

fv_lyme <- fitted( lymefit, newdata=grid1 ,
  summary=FALSE , allow_new_levels=TRUE)
m_lyme <- mean( fv_lyme )
pred_lyme$p <- colMeans( fv_lyme > m_lyme )

lyme_map <-
```

```
  ggplot(pred_lyme) +
  geom_raster(aes(Longitude, Latitude, fill=Probability)) +
  scale_fill_viridis(option="A" ) +
  geom_sf(data=hull, fill=NA, cex=2, color="black") +
  geom_sf(data=states , fill=NA, cex=1 , color="gray") +
  theme_black() +
  theme(panel.grid.minor = element_blank() ) +
  theme(panel.grid.major = element_blank() ) +
  geom_contour( aes( x=Longitude , y=Latitude , z=p) , breaks = c( 0.05 ),
                color="Blue" , cex=1.2, lty=3) +
  geom_contour( aes( x=Longitude , y=Latitude , z=p) , breaks = c( 0.025 ),
                color="Blue" , cex=1.2, lty=2) +
  geom_contour( aes( x=Longitude , y=Latitude , z=p) , breaks = c( 0.005 ),
                color="Blue" , cex=1.2, lty=1) +
  geom_contour( aes( x=Longitude , y=Latitude , z=p) , breaks = c( 0.995 ),
                color="Red" , cex=1.2, lty=1) +
  geom_contour( aes( x=Longitude , y=Latitude , z=p) , breaks = c( 0.95 ),
                color="Red" , cex=1.2, lty=3) +
  geom_contour( aes( x=Longitude, y=Latitude , z=p) , breaks = c( 0.975 ),
                color="Red" , cex=1.2, lty=2)
```

```
newlabels <- c("Distance to River" , "Wetland Area" ,
  "Forest Area" , "Age (years)" , "Male Sex")
gradient_graph<- mcmc_plot(
  gradient_adj, transformations="exp",
  type = "intervals" ,
  variable = c(
    "b_z.River"  ,
    "b_z.Wetland" ,
    "b_z.Forest" ,
    "b_z.Age" ,
    "b_SexM"
  ) )  +
  geom_vline( xintercept = 1 , col = "red" ) +
  scale_y_discrete(labels = newlabels) +
  theme( text = element_text( size = 22 ) ) +
  geom_vline( xintercept = 1 , col = "red" ) +
  geom_hline( yintercept = 7.5 , alpha = 0.3 ) +
  theme_bw() +
  theme( text = element_text(face = "bold" ) ) +
  xlim(0,4.5) +
  xlab("Odds Ratio")
```
